# CD95 promotes metastatic spread via Sck in pancreatic ductal adenocarcinoma

**DOI:** 10.1038/cdd.2014.217

**Published:** 2015-01-23

**Authors:** M Teodorczyk, S Kleber, D Wollny, J P Sefrin, B Aykut, A Mateos, P Herhaus, I Sancho-Martinez, O Hill, C Gieffers, J Sykora, W Weichert, C Eisen, A Trumpp, M R Sprick, F Bergmann, T Welsch, A Martin-Villalba

**Affiliations:** 1 Molecular Neurobiology, German Cancer Research Center (DKFZ), Heidelberg, Germany; 2 Apogenix GmbH, Heidelberg, Germany; 3 Institute of Pathology, University of Heidelberg, Heidelberg, Germany; 4 Division of Stem Cells and Cancer, German Cancer Research Center (DKFZ), Heidelberg, Germany; 5 Heidelberg Institute for Stem Cell Technology and Experimental Medicine gGmbH, Heidelberg, Germany; 6 German Cancer Consortium (DKTK), Heidelberg, Germany; 7 Department of General, Visceral and Transplantation Surgery, Heidelberg, Germany

## Abstract

Cancer stem cells (CSCs) have been implicated in the initiation and maintenance of tumour growth as well as metastasis. Recent reports link stemness to epithelial–mesenchymal transition (EMT) in cancer. However, there is still little knowledge about the molecular markers of those events. *In silico* analysis of RNA profiles of 36 pancreatic ductal adenocarcinomas (PDAC) reveals an association of the expression of CD95 with EMT and stemness that was validated in CSCs isolated from PDAC surgical specimens. CD95 expression was also higher in metastatic pancreatic cells than in primary PDAC. Pharmacological inhibition of CD95 activity reduced PDAC growth and metastasis in CSC-derived xenografts and in a murine syngeneic model. On the mechanistic level, Sck was identified as a novel molecule indispensable for CD95's induction of cell cycle progression. This study uncovers CD95 as a marker of EMT and stemness in PDAC. It also addresses the molecular mechanism by which CD95 drives tumour growth and opens tantalizing therapeutic possibilities in PDAC.

Recent analysis of the cellular heterogeneity within the tumour mass revealed the existence of cells that share characteristics with stem cells of the tissue of origin.^1^ These cells are responsible for the tumour's resistance to current therapies and therefore provide new perspectives in cancer treatment. Cancer stem cells (CSCs) or tumour-initiating cells (TICs) are characterized by their self-renewal and differentiation capacity, which are assessed by their ability to generate a heterogeneous tumour in immunocompromised mice in serial transplantations.^2^ In pancreatic cancer, those properties were initially shown by cells expressing CD24, CD44 and ESA (epithelial surface antigen).^3^

Pancreatic cancer is the fourth leading cause of cancer-related death in the United States of America.^4^ The highly malignant phenotype of pancreatic ductal adenocarcinoma (PDAC) results from aggressive invasion and early metastatic potential. Epithelial–mesenchymal transition (EMT) is considered to be the first step of metastatic spread. During this process, the tumour cells master the ability to detach from their neighbours and gain motile and invasive properties enabling them to spread via blood or lymph vessels.^5^ As cells undergo EMT, they lose their epithelial features including sheet-like architecture, polarity and E-cadherin expression and gradually gain motility and expression of mesenchymal markers such as N-cadherin, fibronectin and vimentin. Recent studies have uncovered a link between the EMT and the acquisition of stem cell characteristics.^6, 7^ Most growth factors such as TGF-*β*, HGF, EGF, IGF and FGF are known to trigger EMT.^8^ Interestingly, there is growing evidence that the so-called ‘death receptor' CD95 (Fas/Apo-1) behaves like a growth factor receptor in cancer cells.^9, 10, 11^

CD95 was first discovered as the initiator of programmed cell death by forming death-inducing signalling complex (DISC, including Fas-associated death domain, FADD and caspase-8/10) upon stimulation with CD95 ligand (CD95L).^12^ However, mitogen-activated protein kinases (MAPKs), leading to p38, JNK or extracellular signal-regulated kinase (ERK) 1/2 activation, were also reported to be driven by CD95.^13, 14^ In glioblastoma multiforme (GBM), CD95-induced migration depends on the formation of the so-called phosphatidyl-inositol 3-kinase (PI3K) activation complex (PAC),^11, 12^ consisting of the Src family kinase (SFK), Yes and p85, the regulatory subunit of PI3K. PAC components, however, differ between cell types, encompassing also other SFKs or the Syk tyrosine kinase.^15, 16^

Here, we show that the expression of CD95 increases in primary PDACs as compared with non-tumour-bearing pancreas and is higher in metastatic pancreatic cells than in primary PDAC. In CSCs isolated from primary PDAC surgical specimens, the expression of CD95 positively correlates with EMT markers. We also identified Sck as the molecular link between CD95 and activation of the PI3K and MAPK pathways. Neutralization of the CD95L reduces PDAC growth and metastasis. The present study defines CD95 and its downstream signalling pathway components as new targets for PDAC therapy.

## Results

### Analysis of CD95 expression in pancreas cancer

**Figure 1:**
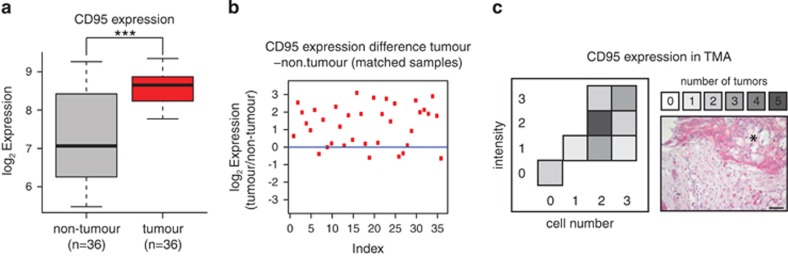
CD95 expression is upregulated in PDAC. (**a**) CD95 expression in matched tumour (*n*=36) and non-tumour samples (*n*=36) from pancreatic cancer patients (***P-value=9.083e−07, Wilcoxon's signed-rank test). (**b**) Ratio of CD95 expression in matched tumour and non-tumour samples from pancreatic cancer patients (GSE15471 data set). (**c**) Analysis of CD95 staining of PDAC sample-derived TMA. Tissue samples were differentially scored for intensity and abundance of CD95-positive tumour cells. Grey scale represents the number of tumour samples. Immunostaining indicates high CD95 expression in tumour tissue compared with surrounding stroma. Asterisk denotes tumour. Scale bar: 50 *μ*m

To study the relationship between CD95 expression and PDAC at a genomic level, we made use of the microarray data set GSE15471 from the GEO database. This data set comprises tumour- and non-tumour-matched samples obtained from 36 pancreatic cancer patients at the time of surgery.^17^ After normalization, we found that CD95 expression is significantly higher in tumour samples compared with non-tumour samples (Figure 1a), and that most patients show an increase in the expression of CD95 as compared with surrounding non-tumour tissue (Figure 1b). To validate these findings, we confirmed the specificity of CD95 and CD95L antibodies according to previous reports (Supplementary Figure 5) and examined a PDAC patient-derived tissue microarrays (TMAs) stained for CD95. A high number and intensity of CD95 immunostaining was exhibited by PDAC tumour cells (Figure 1c). Hence, CD95 expression seems to support the tumourigenic potential of PDAC cells.

### CD95-expressing PDAC cells exhibit EMT properties

**Figure 2:**
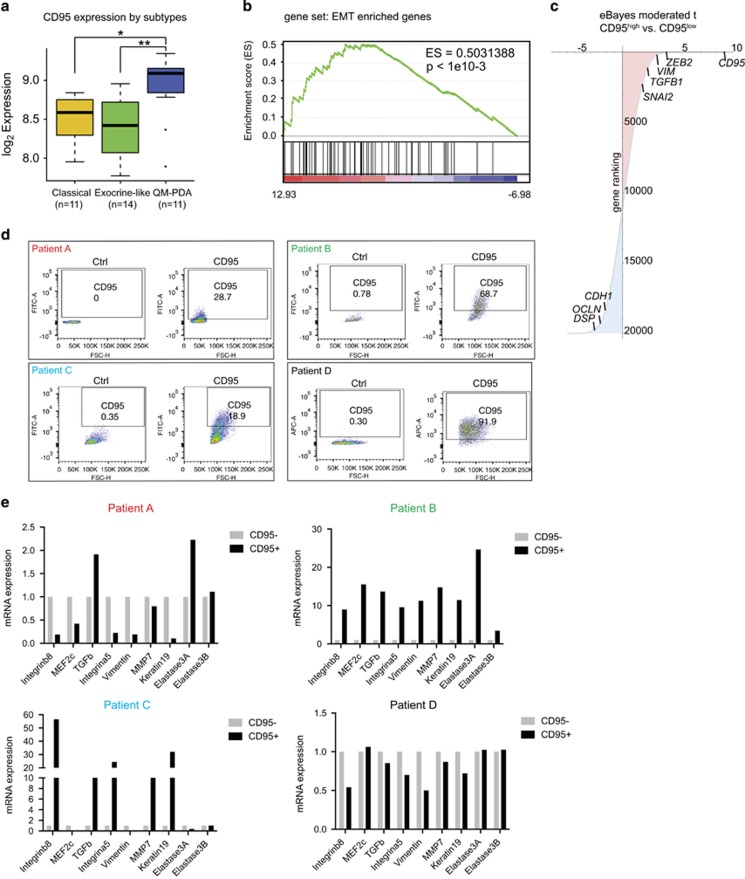
CD95 expression is associated with an EMT signature. (**a**) CD95 expression in tumour samples from pancreatic cancer patients grouped by subtype (classical PDA, *n*=11; exocrine-like PDA, *n*=14; QM-PDA, *n*=11) (*P<0.05, **P<0.01, Wilcoxon's rank-sum test). (**b**) GSEA. Horizontal axis represents ranking of genes according to their differential expression between CD95^high^ and CD95^low^ samples as shown in (**c**). Black bars on the lower part of the plot represent genes from EMT transition gene signature (ES=0.5031388, *P*-value <1e10−3). (**c**) Empirical Bayes moderated t-statistics of differential expression between groups of CD95 expression high and low. Known EMT (*ZEB2*, *VIM*, *TGFB1* and *SNAI2*) and differentiation (*DSP*, *OCLN* and *CDH1*) genes are highlighted. CD95 is also highlighted scoring the highest in the ranking. (**d**) CD95 expression in PDAC-CSC lines derived from four patients (A–D) as measured by flow cytometry. (**e**) mRNA expression analysis of EMT and pancreatic differentiation markers from PDAC-CSC lines derived from Patients A–D. CD95-positive and -negative cells were FACS (fluorescence-activated cell sorting) sorted and mRNA was extracted for quantitative PCR (qPCR) analysis

Next, we compared CD95 expression in pancreas cancer subtypes as defined by Collisson *et al.*^18^ The highest CD95 expression was detected in the *Quasimesenchymal-PDA* subtype as compared with the *Classical-* and *Exocrine-like-PDA* subtypes (Figure 2a). This finding suggested that tumours expressing high levels of CD95 exhibit a mesenchymal phenotype, which led us to study the relationship between CD95 and EMT in more detail. To this end, the tumour samples were divided into three groups of the same size according to their CD95 expression (high, intermediate and low), and preranked genes by their differential expression between CD95 high- and CD95 low-expression samples (Figure 2c). In addition, we applied gene set enrichment analysis (GSEA)^19, 20^ and observed an enrichment of EMT genes^21^ in the CD95 high-expression group (Figure 2b). Next, we aimed to verify the *in silico* results using primary cell lines, which were isolated from four patient-derived xenografts (Figures 2d and e). CD95 expression displayed a wide range from 18% to over 90% of CD95-positive tumour cells (Figure 2d). The variety of CD95 expression in PDAC cells was not because of culture conditions, as freshly isolated tumour cells also showed marked differences in CD95 expression (Supplementary Figure 1). Next, we sorted CD95-positive and -negative cells by flow cytometry and analysed EMT gene expression in the respective populations. Interestingly, PDAC CD95 high-expressing cells from Patients B and C showed high expression of genes characteristic of mesenchymal as well as epithelial identity (Figure 2e). CD95 high-expressing cells from Patient A showed a less pronounced signature; however, a well-characterized trigger of EMT, TGF-*β*, clearly correlated with CD95 expression. Patient D-derived cells did not show an obvious trend, which can be potentially explained by the overall high expression of CD95 in this PDAC line (Figures 2d and e).

### CD95-expressing PDAC cells generate tumours in xenograft models

**Figure 3:**
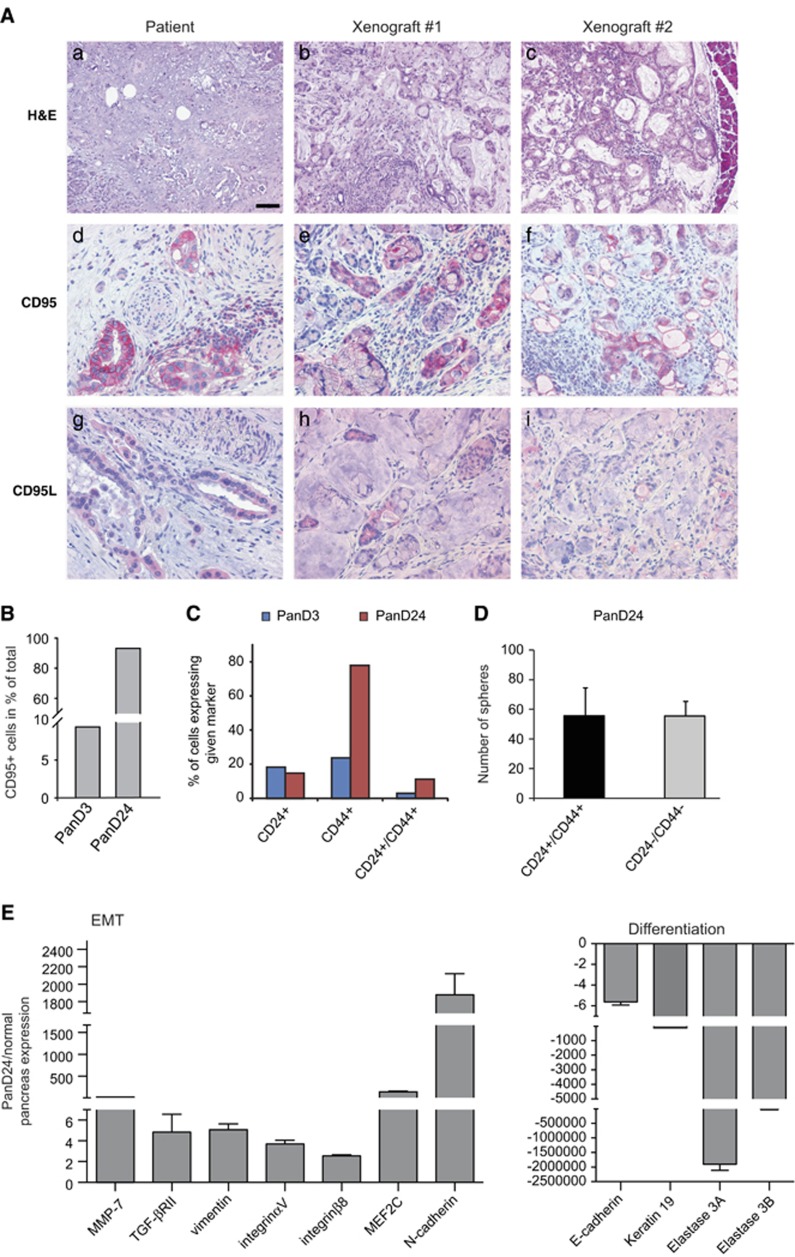
Isolated primary PanD24 cell line exhibits EMT signature. (**a**) Representative images of PanD3-derived PDAC from two xenotransplants (xenografts 1 and 2) and the original human tumour (patient). Representative pictures of hematoxylin and eosin (H&E)-stained and anti-CD95- and anti-CD95L-stained sections are presented. Scale bar: 50 *μ*m. (**b**) Surface expression of CD95 in the primary PDAC-derived PanD3, D24 (measured at passage 2) measured by flow cytometry. (**c**) Cell surface expression of CD24 and CD44 measured via flow cytometry in two PDAC-CSC lines: PanD3 (passage 5) and PanD24 cells (passage 9). (**d**) Tumour sphere assay was performed with FACS (fluorescence-activated cell sorting) sorted PDAC-CSC PanD24 cells (CD24^+^/CD44^+^ or CD24^−^/CD44^−^). Results are expressed as mean±S.D. (**e**) Relative mRNA levels of EMT and pancreatic markers in PanD24 cells and normal human pancreas. Results are expressed as mean of biological duplicates±S.E.M.

To expand the analysis on CD95-expressing PDAC-CSCs, we extracted two cell lines from surgical PDAC specimens and cultured them in stem cell-selective medium. The two cell lines PanD3 and PanD24 exhibit notable differences in CD95 expression, confirming data from other cell lines and freshly isolated tumour cells (Figure 3b). To determine the tumour-initiating capacity of these isolated cell lines, 2.5 × 10^5^ cells were injected into the pancreatic head of Fox Chase SCID Beige mice. Animals injected with PanD3 cells (containing 9.3% of CD95+, 17.8% CD24+ and 23.6% CD44+ cells; Figures 3b and c) developed tumours that were manually detectable 3 months after the injection. PanD3 tumours were strikingly similar to the patient's original tumour and relatively differentiated (Figure 3a). Immunohistochemically, both human and mouse tumours displayed a membrane-bound and cytoplasmic expression of CD95 and CD95L in ductal cells.

PanD24 clone contained much higher proportion of CD95- (93.3%) and CD44-positive (77.6%) cells than other isolated clones (Figures 3b and c). Despite the fact that the combination of CD24 and CD44 expression has been reported to label CSCs, there was no difference in self-renewal properties between CD24+/CD44+ and CD24−/CD44− cells (Figure 3d). A total of 2.5 × 10^5^ PanD24 cells injected into the pancreatic heads of Fox Chase SCID Beige mice formed tumours that were detected by PET scan 21 days after injection (Supplementary Figure 1). These tumours were less differentiated than the patient's original tumour, suggesting the occurrence of EMT (data not shown).

EMT often correlates with metastasis^22, 23^ and stem cell-like characteristics^6^ in PDACs and other solid tumours. The high levels of CD95 as well as poor differentiation of the tumour cells in PanD24 xenografts suggest that this clone underwent EMT. Along this line, several EMT markers were upregulated in PanD24 cells as compared with normal pancreas (Figure 3e). In addition, decreased expression of E-cadherin, keratin-19 and elastases indicates that PanD24 cells lost properties of the differentiated exocrine pancreas (Figure 3e). Also, protein levels of E-cadherin were much lower than in PANC-1 cells (Supplementary Figure 4). PANC-1 cells exhibit a significantly lower expression of CD95 (Supplementary Figure 1), which further supports the assumption that expression of CD95 impinges in the EMT state of the cell. Along this line, stimulation of CD95 increased the protein levels of vimentin in PANC-1 cells but not in PanD24 at the time points tested (Supplementary Figure 4). To test whether CD95 activation also impacted the levels of additional mesenchymal markers, we also attempted to examine N-cadherin and fibronectin levels. However, unlike 3T3 or 293T cells, PANC-1 did not express any detectable levels of N-cadherin and fibronectin. Interestingly, knockdown (KD) of SCK in Panc1 cells decreased CD95-mediated upregulation of vimentin and E-cadherin protein levels, yet a mild induction of vimentin is still detectable (Supplementary Figure 4). Altogether, these data suggest that surface expression of CD95 could be used to isolate highly metastatic CSCs from the tumour's primary material for further screening of anticancer drugs.

### The CD95/CD95L system has a crucial role in tumour growth and metastasis of human PDAC-CSCs *in vivo*

**Figure 4:**
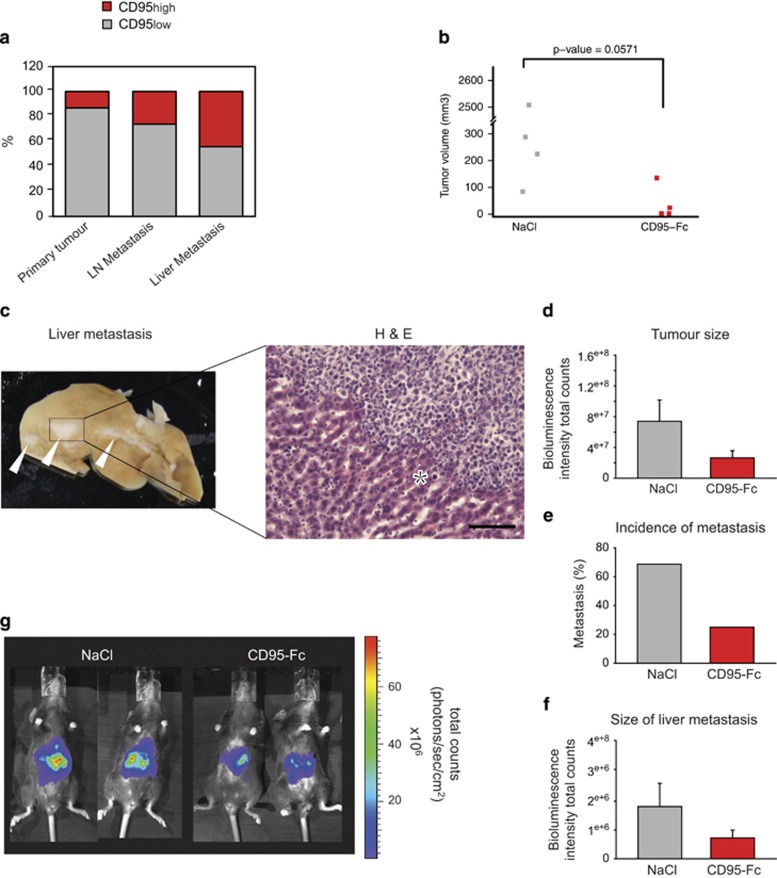
Blocking of CD95L reduces tumour size and metastasis of PDAC. (**a**) Semiquantitative analysis of CD95 immunostaining in TMA containing PDAC samples, and lymph node and liver metastases from different primary PDAC tumours. (**b**) Mice were orthotopically transplanted with PanD24 cells and at days 3 and 7 after transplantation animals were treated with 50 *μ*g CD95-Fc i.p. After 105 days, mice were killed and tumour volumes were assessed with a caliper. Treatment with CD95-Fc (*n*=4) clearly reduced tumour volumes compared with the NaCl- (*n*=4) treated control group (Wilcoxon's rank-sum test, *P*-value=0.05714). (**c**) Picture of a liver from a control mouse with macroscopic liver metastasis, indicated by white arrows (left side), and a representative picture of hematoxylin and eosin (H&E)-stained liver metastasis (right side). No metastatic lesions were detected in the livers of mice treated with CD95-Fc. Asterisk denotes liver tissue. Scale bar: 20 *μ*m. (**d**–**g**) Mice were orthotopically injected with Panc02 cells and at days 3 and 7 after injection animals were treated with 50 *μ*g CD95-Fc (*n*=16) or NaCl (*n*=14) intravenously. Three weeks later, mice were killed and tumour size (**d**) and liver metastases (**f**) was assessed by bioluminescence imaging. (**e**) Percentage of animals with detectable liver metastases. (**g**) Representative bioluminescence pictures. Results are expressed as mean±S.E.M.

We next examined CD95 expression in a TMA-containing samples from primary PDAC tumours, lymph node metastasis and liver metastasis. Analysis of the array revealed a gradual increase of cells expressing CD95 from the primary tumour (13.2%) to the metastatic lesions in the lymph node (26.3%) and liver (44.5%), suggesting that CD95 has a role during PDAC metastatic progression (Figure 4a).

To translate these findings into a treatment regimen, a CD95-Fc fusion protein^24^ was used to block endogenous CD95L–CD95 interaction. PanD24 cells were injected into the pancreas head of Fox Chase SCID Beige mice. Three and seven days after the orthotopic injection, mice were intravenously treated with either CD95-Fc or NaCl and monitored until the establishment of palpable tumours 105 days later. At this time point, tumours were bigger in the saline- than in the CD95-Fc-treated group (Figure 4b). Additionally, only one mouse in the saline-treated group and none in the CD95-Fc-treated group exhibited macroscopically visible liver metastasis (Figure 4c).

To address the function of CD95 in metastatic tumour cells, we used the Panc02 syngeneic mouse model. C57BL/6 mice were treated 3 and 7 days after orthotopic injection of Panc02 cells (containing 93% of CD95-positive cells, data not shown) with CD95-Fc and killed 21 days later. Animals treated with CD95-Fc showed reduced tumour volumes compared with the saline-treated ones (Figures 4d and g). Furthermore, treatment with CD95-Fc reduced the incidence of liver metastasis and the size of metastatic tumours as compared with untreated animals (Figures 4e and f). Taken together, our findings demonstrate that the protumourigenic effects of the CD95/CD95L system can be overcome by blocking endogenous CD95L.

### CD95 activates PI3K and MAPK/ERK pathway via Sck

**Figure 5:**
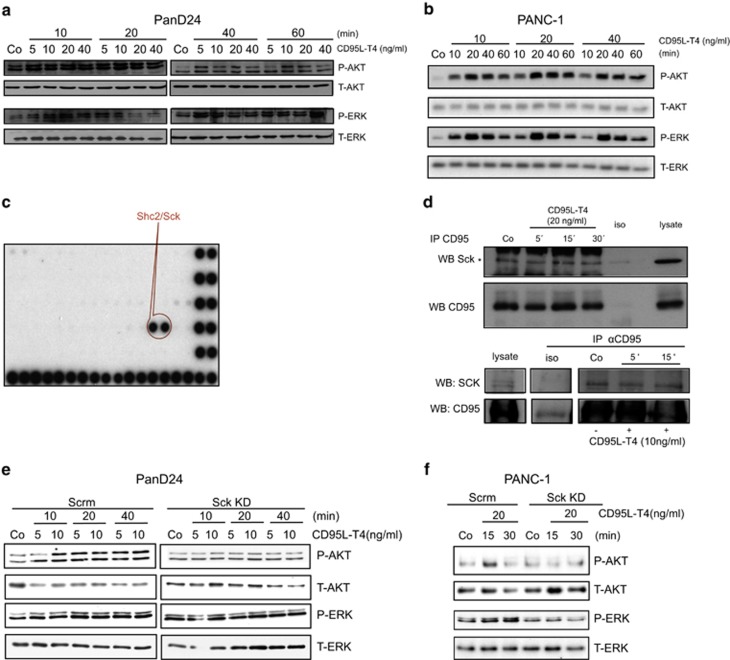
Sck binding is required for CD95-induced PI3K/MAPK cascades. (**a**) Phosphorylation of AKT and ERK upon treatment with the indicated doses of CD95L-T4 for the different time points is shown in PanD24 cells. (**b**) Phosphorylation of AKT and ERK upon treatment with the indicated doses of CD95L-T4 for the different time points is shown in PANC-1 cells. (**c**) TranSignal SH2 domain arrays showing binding of endogenous CD95 to the SH2 domain of Sck in PANC-1 cells. (**d**) Co-immunoprecipitation of CD95 and Sck is shown in PANC-1 cells. Increased recruitment of Sck is observed after 15 min stimulation (upper panels). CD95 was immunoprecipitated from PanD24 cells treated with CD95L-T4 for 5 or 15 min. The immunoprecipitates were immunoblotted with anti-Sck antibody (lower panels). Data are representative of two independent experiments. (**e** and **f**) PanD24 (**e**) and PANC-1 (**f**) cells were transiently transfected with either non-targeting siRNA (Scrm) or Sck-siRNA (Sck KD) upon treatment with the indicated doses of CD95L-T4 and different time points

PanD3 and PanD24 clones are resistant to CD95-induced apoptosis, even at high doses of CD95L-T4,^11^ a recombinant trimerized CD95L (Supplementary Figure 1). In previous reports, we have unravelled the promigratory/invasive molecular underpinnings of CD95 signalling both in pathological^11, 16^ and physiological^15^ scenarios. As pharmacological inhibition of CD95 affects both metastasis and proliferation *in vivo*, we examined the molecular mechanism leading to the latter phenomenon. Stimulation of PanD24 cells with CD95L-T4 induced both AKT and ERK phosphorylation and led to inhibition of GSK3*β* as evidenced by Ser9 phosphorylation (Figure 5a and Supplementary Figures 2 and 3). Along this line, we further examined the activation of the AKT/GSK3*β* and ERK pathway in an established pancreatic cell line (PANC-1), which expresses CD95 (48%) and is known to be resistant to CD95-induced apoptosis (Supplementary Figure 1).^25^ We observed a similar effect in this established cell line, indicating a conserved mechanism (Figure 5b and Supplementary Figures 2 and 3). Activation of the AKT/GSK3*β* and ERK pathway exhibited a concentration- and time-dependent bell-shaped response that has been previously shown in glioma cells^11^ and discussed elsewhere.^12^

We further explored the upstream events of CD95 signal transduction to find a convergent point of PI3K and MAPK pathway regulation. First, the role of DISC components was addressed via KD of FADD, the only known adaptor for death effector domain-containing proteins such as caspase-8. Caspase-8 was recently shown to interact with p85 subunit of PI3K.^26^ FADD removal, however, did not lead to the impairment of PI3K or MAPK pathways, but rather resulted in their slight acceleration as phosphorylation of ERK and AKT appeared at earlier time points than in cells transfected with non-targeting siRNA (Supplementary Figure 3). After excluding the role of DISC components, we decided to focus on K-Ras, a guanine exchange factor frequently mutated in PDACs. As expected, the phosphorylation of ERK was completely abolished upon K-Ras KD. Surprisingly, phosphorylation of AKT was still present (Supplementary Figures 2 and 3). These results indicate that in pancreatic cancer cells K-Ras has an important role in the activation of the MAPK/ERK pathway but not in the initiation of the PI3K pathway.

The ITAM/ITIM-like motif surrounding Tyr291 of CD95^12, 27^ offers an alternative-docking site for the allocation of SH2-containing proteins. Thus, we sought for the identification of novel CD95 interaction partners by using a proteomic screening approach. After incubation of CD95L-stimulated cell lysates of PANC-1 and PanD3 cells with SH2 domain protein arrays, showed the spots corresponding to the SH2 domain of Shc2/Sck demonstrated the strongest CD95 binding (Figure 5c and Supplementary Figure 2). Sck is a well-known adaptor molecule binding to receptor tyrosine kinases via its SH2 domain. The association of Sck with CD95 in unstimulated and CD95L-treated Panc1 and PanD24 cells was confirmed by immunoprecipitation of CD95 and expression analysis of Sck by western blot (Figure 5d). To confirm the involvement of Sck in CD95 signalling, we performed KD experiments. The KD efficiency was confirmed by qPCR (Supplementary Figure 2). Under those conditions, CD95-dependent phosphorylation of both AKT and ERK was abolished in PanD24 and PANC-1 cells (Figures 5e and f and Supplementary Figure 3).

### CD95 stimulation leads to cell cycle progression and induces migration in a Sck-dependent manner

**Figure 6:**
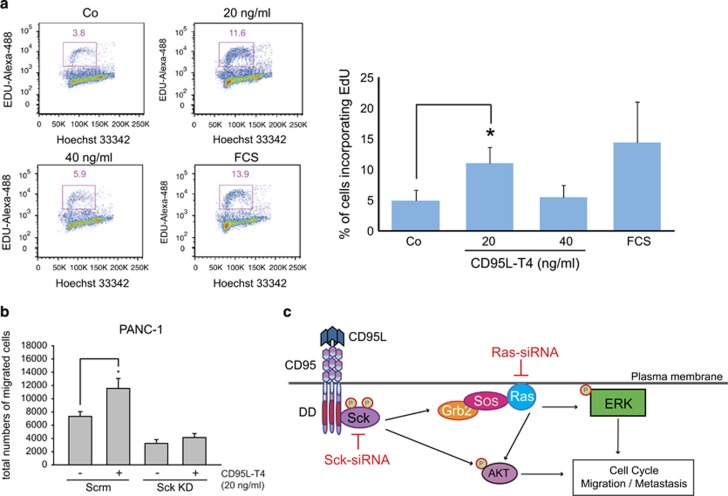
CD95 stimulation leads to cell cycle progression and Sck-dependent migration. (**a**) In PANC-1 cells, DNA replication was assessed after 2 h of incorporation with EdU. EdU was labelled with Alexa-488 and detected by Click-iT assay. DNA was stained with Hoechst. Results are expressed as means of three independent experiments±S.D. (*P<0.05). Lower panels show representative histograms of Alexa-488/Hoechst staining. (**b**) CD95-induced migration is abolished upon Sck KD in PANC-1 cells. Results are expressed as mean of biological duplicates±S.E.M. (*P<0.05). Co, untreated cells; Iso, isotype control; *, specific band. (**c**) Stimulation of CD95 receptor with CD95L leads to the recruitment of Sck and activation of PI3K/ERK pathways resulting in cell cycle progression

AKT and ERK signalling have previously been indicated as mediators of proliferation in cancer cells. Therefore, we aimed to investigate whether CD95 activation would increase the proliferation of pancreatic cancer cells. PANC-1 cells were treated with CD95L-T4 and ethynyl deoxyuridine (EdU), a thymidine analogue. EdU incorporation reflects DNA replication. Cells treated with 20 ng/ml of CD95L-T4 contained a higher fraction of EdU-positive cells as compared with the untreated sample (Figure 6a), thus confirming accelerated entry into the S phase. To further prove the function of Sck, we performed Sck-KD in PANC-1 cells to see whether Sck has an impact on CD95-induced migration. Interestingly, Sck KD suppressed CD95-dependent migration of tumour cells *in vitro* (Figure 6b). These data indicate that Sck serves as a functional link between CD95 and PI3K/MAPK pathways in PDAC. Taken together, we demonstrated that CD95 activation leads to cell cycle progression through the recruitment of Sck, which is indispensable for CD95-dependent migration/metastasis formation (Figure 6c).

## Discussion

In this study, we show that CD95 expression strongly correlates with stemness and EMT and demonstrates that CD95 drives migration and proliferation in PDACs. Moreover, blocking the receptor *in vivo* decreases tumour growth and metastasis. We also identified an adaptor protein, Sck, as an indispensable component of the signalling pathway involving PI3K and MAPK activation that drives this process.

CD95 was initially described as an inducer of apoptosis and thus considered to be a tumour suppressor. However, recent studies in animal models of cancer substantiate the notion of CD95 as a protumourigenic signal.^28^ In this work, we assessed the role of CD95 in PDAC growth/metastasis *in vivo*. Animals injected with a murine PDAC cell line rapidly developed tumours as well as liver metastases. Most importantly, both tumour and metastases formation were successfully halted by blocking CD95L with CD95-Fc. This model has an advantage of using animals with functional immune system, as inflammation is an important factor in pancreatic cancer progression.^29^ Moreover, these findings were reproduced in a xenograft model. PanD24 cells exhibited an EMT signature despite the fact that no metastases were detected at the time of patient's surgery. Thus, this cell subset might have already possessed invasive potential as the EMT programme was shown to be induced in highly metastatic pancreatic cells selected *in vivo*.^22^ In addition, it was recently demonstrated that a subset of pancreatic cells undergoes EMT, enters the bloodstream and seeds the liver before PDAC fully develops,^7^ which might explain the detection delay of the patient's metastasis. The relevance of CD95 expression for detection of EMT is further supported by the fact that CD95-positive cells within a tumour exhibit a higher concomitant expression of epithelial and mesenchymal transcripts as compared with CD95-negative tumour cells. Based on the findings of Mani *et al.*,^6^ we can conclude that the presence of EMT markers in PanD24 also indicates their stemness.

The ‘hunt' for CSC markers to isolate and characterize these cells promises a deeper understanding of their biology. However, these markers do not serve as therapeutical targets.^30^ Here, we show that CD95 expression in pancreatic cancer correlates with stemness, and therefore CD95 might also be considered as a CSC marker. More importantly, this receptor serves as a potential therapeutic target as CSC ability to self-renew and metastasize is impaired after treatment with CD95-Fc.

Although CSCs and EMT cells share common features, several recent studies report that CSCs/TICs are highly proliferative,^31^ which contradicts the classical interpretation of cells that underwent EMT. However, development of more sophisticated high-throughput techniques has revealed marked heterogeneity within tumours on genetic as well as transcriptional level.^32^ Hence, it is plausible to believe that a similar heterogeneity exists among tumour-derived mesenchymal cells. Along these lines, a recent study of circulating breast tumour cells observed the appearance of multicellular tumour-derived clusters expressing mesenchymal markers.^33^ The authors speculate that these clusters could, similarly to the PanD24 cells, result from the proliferation of single tumour-derived mesenchymal cells.

On the molecular level, we dissected the signalling events involved in AKT-dependent cell cycle progression, albeit without excluding the role of MAPKs. We have also elucidated a novel component and properties of PI3K kinase activation complex (PAC).^11, 15, 16^ Tyr291 present in CD95 death domain has been shown to undergo phosphorylation upon receptor activation via number of the SKFs.^11, 27^ In addition, caspase-8 also contains a similar SH2-binding motif (YXXM), and caspases were found to activate MEKK-1 (MAPK/ERK kinase kinase 1) via proteolytic cleavage.^34^ However, as FADD KD affects neither AKT nor ERK phosphorylation, DISC components (i.e. FADD and caspase-8) appear to be dispensable for PAC formation. Those results stand in accordance with the observations made for lpr^cg^ mice, carrying spontaneous CD95 mutation (I225N) that prevents both FADD binding and induction of apoptosis.^35^ This mutation should not affect the ITAM-like motif of CD95, thus still allowing PAC formation. Partial hepatectomy leads to liver regeneration in lpr^cg^ mice in contrast to lpr mice (that do not express CD95 on the cell surface).^36^ Moreover, lpr^cg^ mice develop liver tumours when transplanted with wild-type bone marrow. Taken together, those results suggest that tumour-promoting/proliferative potential of CD95 is not dependent on the DISC formation. The performed SH2 array uncovered Sck/Shc2 as a novel PAC member and signal transducer in PDAC. Interestingly, it was reported that blocking Shc/Grb2 interaction suppressed the growth of B104-1-1 tumours xenografted in nude mice,^37^ showing that cancer treatment might also target the adapter proteins.

The treatment of PanD24-transplanted animals with CD95-Fc resulted in decreased tumour growth strengthening the conclusions derived from murine tumour treatment. CD95-Fc is already in the phase II of clinical trial for GBM treatment, in which the combination of a drug with radiotherapy was compared with stand-alone radiotherapy.^38^ Strikingly, the study objective of increasing the percentage of patients reaching the 6-month rate of progression-free survival by 100% in the CD95-Fc group was substantially exceeded. The findings presented in this manuscript open the possibility to extend such treatment to pancreatic cancer patients. In addition, we identify a specific conductor of CD95-elicited tumourigenic signal, namely Sck that could serve as additional therapeutic target.

## Materials and Methods

### Analysis of microarray data

Microarray data set GSE15471 from GEO database^39^ consists of 39 pairs of surrounding and tumour tissue samples from pancreas of 36 pancreatic cancer patients (there are technical replicates for three of the pairs). Raw data were normalized by the RMA method with the Bioconductor package *affy* in R.^40^ Technical replicates were removed. Probe sets corresponding to FAS (CD95) were mapped to Symbols using the version 2.6.3 of the Bioconductor package *hgu133plus2.db.* In each array, they were summarized taking the maximum value.

Statistical significance of the difference in CD95 expression between matched tumour and non-tumour samples was assessed with paired Wilcoxon's signed-rank test. Differences in CD95 expression between pancreas cancer subtypes were assessed by doing all pairwise comparisons with Wilcoxon's rank-sum test and correcting for multiple testing with the Holm method. Both analyses were performed in R.

GSEA was as described in Mootha *et al.*^19^and Subramanian *et al.*^20^ Probe sets were preranked according to the differential expression between CD95 High (33% of samples with highest CD95 expression) and CD95 Low (33% of samples with lowest CD95 expression) tumour samples using empirical Bayes moderated t-statistics^41^ computed with the limma package^42^ from Bioconductor (‘R package' UCR, Institute for integrative genome biology, Riverside, CA, USA). Probeset values were summarized in a per gene basis mapping to Symbols using the version 2.6.3 of the Bioconductor package *hgu133plus2.db* and taking the most significant per gene.

We used the ‘EMT transition gene signature' from Anastassiou *et al.*^21^ The ‘intestinal stem cell gene signature' was generated by inclusion of the human homologues from the intestinal ‘mRNA stem cell signature' published in the Supplementary Table S3 from Munoz *et al.*^43^ using the Biomart tool (Ensembl v.69; Flicek *et al.*^44^).

### Primary cell lines

To PDAC cultures from Patients A–D (Supplementary Table S1), tumours were cut into pieces of 1–2 mm^3^ and implanted onto the pancreatic body of NOD.Cg-*Prkdcscid Il2rgtm1Wjl* (NSG) mice. Primary xenografts were resected after attaining a volume of ~1 cm^3^. Tumour pieces were dissociated into single cells by incubation with 1 *μ*g/ml collagenase IV (Sigma, St. Louis, MO, USA) for 2 h at 37 °C. The resulting suspension was filtered through a 100 *μ*m mesh and cell debris and dead cells were removed by density centrifugation (FiColl Paque Plus; Amersham, Glattbrugg, Switzerland). For establishing cultures, 5 × 10^6^ cells were seeded into T75 flasks in serum-free medium as described earlier.^45^ Adherent monolayer cultures were maintained at 37 °C and 5% CO_2_. After outgrowth of tumour cells, contaminating fibroblasts were removed by differential trypsinization. All human tissue samples were obtained with written informed consent under protocols approved by the review board of the Medical Faculty of the University of Heidelberg (Heidelberg, Germany).

The lines PancD3 and PanD24 were isolated from tumour samples after resection of the PDAC at the Department of General, Visceral and Transplantation Surgery, University of Heidelberg. The study was conducted in accordance with the Declaration of Helsinki. The specimen collection was approved by the ethical committee of the University of Heidelberg (votes 301/2001 and 159/2002) and informed consent was obtained from the patients. The human primary PDAC-CSCs were cultured in DMEM, 50% F12 supplement, 2% B27 supplement (Invitrogen, Life Technologies GmbH, Darmstadt, Germany), 0.5 *μ*g/ml insulin, 2 nM progesterone, 10 *μ*M putrescine, 3 nM selenium dioxide, 0.1 mg/ml bovine apo-transferrin (all from Sigma-Aldrich, St. Louis, MO, USA), 1% Pen/Strep, 20 ng/ml bFGF (ReliaTech, Wolfenbüttel, Germany) and 20 ng/ml EGF (Promocell, Heidelberg, Germany).

### Orthotopic injection into the pancreas

Eight-to-ten-week-old female C57Bl6A or SCID beige mice were used for orthotopic implantation of Panc02 (1 × 10^4^ cells) or CSC (2.5 × 10^5^ cells), respectively. Panc02 cells were stably infected with a luciferase containing lentiviral vector. Saline or CD95-Fc (50 *μ*g) were applied i.p. 3 and 7 days after transplantation. All animal experiments were performed in accordance with institutional guidelines of the DKFZ and approved by the Regierungspräsidium Karlsruhe.

### TMA analysis

TMA were prepared from formalin-fixed, paraffin-embedded donor blocks. Core tissue biopsy specimens (diameter 1.5 mm) from representative tumour areas and tissue biopsy specimens of nonneoplastic pancreatic parenchyma were taken. For quality control, sections were reviewed and approved by two pathologists (FB and WW). TMA from Figure 1c contained 20 PDAC samples derived from 10 different patients. CD95 staining was scored semiquantitatively from 0 to 3. Scores for the number of cells and intensity of staining were differentially assessed. TMA from Figure 4a comprised 37 different PDAC samples, 17 lymph nodes and 10 liver metastases from different primary PDAC tumours metastases. CD95 staining was performed using *α*-APG101 antibodies (Apogenix GmbH, Heidelberg, Germany) and subsequently scored semiquantitatively by two of the authors, from 1 to 3 taking into account the number of cells and intensity of staining in a blinded manner. Results were averaged and grouped in CD95 low (<1) and high (≥1) samples.

### Immunohistochemistry staining

Immunohistochemistry staining of paraffin-embedded pancreatic tumour sections was performed as described previously^11^ using the following antibodies: CD95 (*α*-APG101; Apogenix GmbH), CD95L (CD95L; ab15285; Abcam, Cambridge, UK).

## Abbreviations

bFGF: basic fibroblast growth factor
CD95L: CD95 ligand
CSC: cancer stem cells
DED: death effector domain
DISC: death-inducing signalling complex
EdU: ethynyl deoxyuridine
EGF: epidermal growth factor
EMT: epithelial–mesenchymal transition
ESA: epithelial surface antigen
GBM: glioblastoma multiforme
GSEA: gene set enrichment analysis
KD: knockdown
MEKK-1: MAPK/ERK kinase kinase 1
NaCl: sodium chloride
PAC: PI3K activation complex
PDAC: pancreatic ductal adenocarcinoma
PI: propidium iodide
PI3K: phosphatidyl-inositol 3-kinase
SCID: severe-combined immunodeficiency disease
SFK: Src family kinase
Srcm: scrumbel
TIC: tumour-initiating cell
TMA: tissue microarray
