# Supplementary material for: CD95 promotes metastatic spread via Sck in pancreatic ductal adenocarcinoma
Source: Cell Death Differ. 2015 Jan 23;22(7):1192–202. doi: 10.1038/cdd.2014.217 (PMC4572867; doi:10.1038/cdd.2014.217)
Supplement: Supplementary Information [file cdd2014217x1.doc]

SUPPLEMENTAL INFORMATION

**Patient tissue samples for the tissue micro array (TMA)**

Tumor samples (pancreatic tissue and metastases) for the present study were randomly selected from patients that underwent pancreatic resection for PDAC or resection of liver metastases between March 2002 and September 2004 at the Department of Surgery, University of Heidelberg, Germany. Normal pancreatic tissue samples were from pancreatic resections for focal lesions of the pancreas that did not extend throughout the entire specimen. Informed consent was obtained preoperatively from the patients in accordance with the Helsinki Declaration. Tissue sampling and analysis was approved by the local ethical committee of the University of Heidelberg (approval number 301/2001).

Diagnoses were established according to World Health Organization (WHO) grading.

**Positron emission tomography** (**PET**)

In preparation for the scans, the mice were anesthetized with sevoflurane and then maintained under anesthesia during the scan (2.5% induction and 1% maintenance). 18-F FDG was synthesized in the Department of Radiochemistry (German Cancer Research Center, Heidelberg, Germany). The plasma glucose level of the animals was determined using a blood glucose sensor electrode (MediSense, Waltham, MA, USA). A transmission scan was done for 10 minutes prior to tracer administration with two rotating germanium pin sources to obtain cross sections for attenuation correction. PET data were acquired for 1 hour in list mode on a Siemens Inveon scanner (Siemens Erlangen Germany) using a matrix of 256 x 256 (pixel size 0.3882 x 0.3882 x 0.796 mm). Therafter images were reconstructed at definite time periods after administration of 5-10 MBq ^18^FDG (2 x 15 s, 8x30 s, 5x60 s, 4x120 s, 2 x 210 s, 7x300 s). The images were reconstructed iteratively using the space alternating generalized expectation maximization method (SAGE, 16 subsets, 4 iterations) applying median root prior correction and were converted to standardized uptake value (SUV) images on the basis of the formula “SUV = tissue concentration (Bq/g) / (injected dose [Bq]/ body weight [g])”. The individual tumour SUV was calculated for each study as the mean of the last two time frames from 50-60 min p.i.

**Cell culture**

PANC-1 were cultured in Dulbecco Modified Eagle’s Medium (DMEM, Sigma-Aldrich), 50% F12 supplement (Gibco), 10% fetal calf serum (FCS, Biochrom AG), 1% penicillin/streptomycin (Pen/Strep, Gibco) and the mouse PDAC cell line Panc02 in DMEM, 10% FCS, 1% Pen/Strep.

**Isolation of RNA and Real-time Quantitative PCR**

RNA was isolated from cultured cells using RNeasy Mini Kit (Qiagen). 2 μg of RNA was reversely transcribed into cDNA by incubating RNA with 2 μl 10x PCR buffer (without MgCl2), 4 μl 50 mM MgCl2, 1 μl RNase inhibitor, 1 μl dNTPs (all reagents purchased from Applied Biosystems), 1 μl 50 mM oligo dT primers (Eurofins MWG Operon) and 10 μl nuclease-free water for 3 minutes at 65°C followed by 5 min incubation on ice. Subsequently 1 μl of 50 U/μl reverse transcriptase was added and the reaction was incubated for 45 minutes at 42°C, followed by 5 min at 95°C and chilled at 4°C.

In all cases, real-time quantitative PCR was carried out using Power SYBR® Green PCR Master Mix (Applied Biosystems) according to manufacturer’s instructions. The following primer pairs were used: Sck (5'-CGAGGTTCTCCGCTCTATGC-3' and 5'-ATGTGGTGGTTGGCGATGAC-3'), K-Ras (5’-ACAAGACAGGGTGTTGATGATGCC-3' and 5'-AGCTAACAGTCTGCATGGAGCAGG-3’) and β-actin (5’-ACCCACACTGTGCCCATCTACGA-3’ and 5’-CAGCGGAACCGCTCATTGCCAATGG-3’, loading control).

For EMT markers, the following primers were used: MMP-7 (5’- ACAGTGGGAACAGGCTCAGGACT-3’ and 5’-TCTGGCACTCCACATCTGGGC-3’), TGF-β RII (5’-GCGCTGGGGGCTCGGTCTAT-3’ and 5’-GGCCTCCATTTCCACATCCGACT-3’), vimentin (5’-TCCCTGGAACGCCAGATGCG-3’ and 5’-GCAGAGAAATCCTGCTCTCCTCGC-3’), integrin αV (5’-TCTGTGCCGCGCCTTCAACC-3’ and 5’-ACATCCGGGAAGACGCGCTG-3’), integrin β8 (5’-TGCCAGGTGCCTTGCGCTG-3’ and 5’-TAGCTTCGGCTCCTGGACGCA-3’), MEF2C (5’-CCTTCATCAGGAACGAATGCAGGAA-3’ and 5’-AGTCACACAGCACGCTCAGCTC-3’), keratin 19 (5’-AGCTGAGCATGAAAGCTGCCT-3’ and 5’-GATCTTCCTGTCCCTCGAGCA-3’), elastase 3A (5’-TTGGGTGAGTACAACCTTGCT-3’ and 5’-CCCATTGGTATAGAGACGGC-3’), elastase 3B (5’-CAGTCAGCCCCCTTTGCTTA-3’ and 5’-CATAGCCTGAGGCAACGGC-3’), E-cadherin (5’-CAGTACAACGACCCAACCCA-3’ and 5’-CACGCTGACCTCTAAGGTGG-3’), N-cadherin (5’-CCAGAAAACTCCAGGGGACC-3’ and 5’-TGTGCCCTCAAATGAAACCG-3’) and 18S rRNA (5’-GTAACCCGTTGAACCCCATT-3’ and 5’-CCATCCAATCGGTAGTAGCG-3’, loading control). Data were analyzed using the 2Δ Ct method.

**Protein Extraction**

PANC-1 cells were grown in a monolayer. Culture medium was replaced with a serum-free medium 12 h before the treatment with CD95L. Cells were treated with the denoted concentrations of CD95L-T4, soluble trimerized human CD95L (Apogenix GmbH)^11^. After the treatment the dishes were washed with cold PBS and then scraped in PBS (with 1mM sodium orthovanadate, 10 mM NaF, 10 mM NaN3, 10 mM p-nitrophenylphosphate (pNPPi), 10mM Sodium pyrophosphate (NaPi), 10 mM β-glycerophosphate and complete protease inhibitor cocktail ((Roche), 1 tablet/50 ml). After 5 min centrifugation cells were lysed in 0.2 % SDS (with 1mM sodium orthovanadate, 10 mM NaF, 10 mM NaN3, 10 mM p-nitrophenylphosphate (pNPPi), 10 mM Sodium pyrophosphate (NaPi), 10 mM β-glycerophosphate and complete protease inhibitor cocktail ((Roche), 1 tablet/50 ml).

Primary cells, grown in suspension, were synchronized with 2 mM thymidine 12 h before CD95L treatment and collected in a similar way as described above.

The protein concentration was determined using the BCA protein assay (Thermo Scientific) by comparing to standardized concentrations of bovine serum albumin (BSA).

**Western Blotting**

Equal amounts of protein from cell lysates (20-50 µg depending on the antibody used for detection) in sample buffer were separated by sodiumdodecylsulphate-polyacrylamide gel electrophoresis (SDS-PAGE) on 10-15 % polyacrylamide gels.

Proteins were transferred from polyacrylamide gels onto nitrocellulose membranes by electroblotting. Blotting was performed at 30V/gel for 1 to 2 hours at 4°C. Following transfer, non-specific binding sites on the nitrocellulose membrane were blocked by incubation with 5 % skim milk powder in PBS-Tween for 1 h. After washing, the membranes were incubated overnight at 4°C with primary antibody (usually diluted in PBS-Tween containing 5 % skim milk powder) on a shaker. Following thorough washing, antibody binding was visualized via horseradish peroxidase (HRP)-conjugated secondary antibodies, with which the membranes were incubated for 1 h. The HRP signal was detected by incubation with ECL solution and consecutive exposure to X-ray films (Hyperfilm, Amersham). Following antibodies were used: Actin (Actin, 1:3000, Santa Cruz Biotechnologies #sc-1616), phosphorylated Akt (P-Akt, 1:1000, Cell Signaling #9271), total Akt (T-Akt, 1:1000, Cell Signaling #9272), E-cadherin (E-cadherin, 1:1000, Cell Signaling #3195), FADD (mouse monoclonal Ab, clone 1F7, 1:1000, Millipore #05-486), phosphorylated ERK (P-ERK, 1:1000, Cell Signaling #4370), total ERK (T-ERK, 1:1000, Santa Cruz Biotechnologies #sc-154), Fibronectin (Fibronectin, 1:20000, BD Biosciences 610077), phosphorylated GSK3β (P-GSK, 1:1000, Cell Signaling #9331), total GSK3β (T-GSK, 1:1000, Santa Cruz Biotechnologies #sc-7291), N-cadherin (N-cadherin, 1:2000, BD Bioscience 610920), Sck (Sck, 1:1000, Santa Cruz Biotechnologies #sc-33807), Vimentin (Vimentin, 1:1000, Santa Cruz Biotechnologies #sc-6260).

**Blot Stripping**

For removal of antibody complexes from nitrocellulose membranes, membranes were subjected to three washes with 1 M Glycine pH 1,8. After thorough washing with PBS-Tween and blocking unspecific binding sites with 5 % skim milk powder, the membranes were reprobed as described above.

**Quantification of Western Blots**

Western blots were quantified with ImageJ software. First, the area surrounding a band was selected with the rectangular tool to draw a plot for each lane. The plot was then utilized to quantify area under each peak. The values corresponding to band intensities (area of the peak) of phosphorylated proteins were divided by their respective loading control values. For each experiment these values were normalized to the untreated control.

**Knock-down Experiments**

Knock-down experiments were performed by transient transfection with Lipofectamine 2000™ (Invitrogen) for PANC-1 cells following the instruction manual.

PDAC-TICs were transfected with Neon Transfection System (Invitrogen). The cells were washed with HBSS w/o Mg2+ and Ca2+. 4x10^6^ cells were resuspended in 100 μl of Buffer R together with 10 μl of siRNA (100 μM stock). The Neon Tube was set up with 3 ml of Buffer E and the electroporation was conducted according to following parameters: single pulse, 1500V, 20 ms width. Three electroporations were pooled and cultured in the medium without antibiotics.

All the experiments were performed using ON-TARGETplus SMARTpool validated siRNAs against K-Ras, Sck, CD95 or FADD (Dharmacon/ThermoFisher: K-Ras, L-005069-00-0010; Sck, L-031192-00; CD95, L-003776-00; FADD, L-003800-00), and a non-targeting pool of siRNAs as a negative control to exclude off-target effects (Dharmacon/ThermoFisher, D-001810-10-05). After transient transfection with the different siRNAs, cells were cultured for 72h before being treated with CD95LT4 (20ng/ml). Knock-down efficiency was controlled by quantitative real-time PCR.

**Migration Assay**

Migration of PANC-1 cells was assessed *in vitro* in a two-chamber migration assay after efficient knock-down of Sck (48h). Transwell inserts [8 µm (BD #353097) pore size] were coated with collagen, type I (50 μg/ml; Promocell). 1 x 10^5^ cells were plated in 300 µl medium onto the upper chamber. Cells were left untreated or treated with CD95L-T4 20 ng/ml in the upper chamber. The number of migrated cells was counted 36 hours after treatment.

**Quantification of DNA replication**

DNA replication was assessed by using Click-iT® EdU Cell Proliferation Assay (Invitrogen) according to manufacturer’s instructions. EdU was labeled with Alexa-488 and detected by Click-It kit. DNA was stained with Hoechst. Results are expressed as means of biological triplicates ± SD (*p<0.05). Lower panels show representative histograms of Alexa-488/Hoechst staining.

**PI cell cycle assay after CD95 stimulation**

G1/S-phase arrest was achieved by treating PanD24 cells with 2mM Thymidine (dissolved in PBS) in PDAC primary medium for 12 hours, subsequent release from Thymidine for 12 hours and again 12 hours 2mM Thymidine treatment. Cells were washed once with PBS and 2x10^5^ were seeded in 1ml of PDAC primary medium in a 12-well plate and subsequently treated with 5 and 10ng/ml of CD95L-T4 or left untreated. Following incubation for 4 hours at 37°C and 5% CO_2_, cells were harvested and centrifuged for 5 minutes at 600g. For each condition 3 biological replicates were used. The cells were resuspended in 45µl of cold 1xPBS. They were fixed by adding 105µl of 100% ice-cold Ethanol and stored at -20°C until further proceeding. For staining with Propidium iodide (PI) cells were washed with 200µl cold PBS and subsequently treated with 50µl of RNase A (diluted in PBS to 50µg/ml, Fermentas) at room temperature. After centrifugation at 1350g for 5 min, 50µl of PI buffer was added and incubated for at least one hour. The stained cells were analyzed by flow cytometry. The fraction of cells in each phase of the cell cycle was determined by means of the FlowJo® software by gating on living single cells and subsequent cell cycle analysis.

**Flow cytometry**

For all stainings, cells were resuspended in FACS buffer (PBS, 10% FCS) and kept for 10 minutes on ice before stained with the respective antibodies (PE mouse anti-human CD24, FITC mouse anti-human CD44, APC mouse anti-human CD44, all from BD Bioscience, APC/Cy7 anti-human CD45 from Biolegend and APC rabbit anti-human Apo1 kindly provided by Apogenix GmbH). Cells were incubated for 1 h on ice, washed 2 times with FACS buffer and once with PBS afterwards. Samples were run on a FACSCanto II flow cytometer (BD) and analyzed using FACSDiva™ (BD) software or FlowJo software.

**SH2 Array**

The TranSignal SH2 Domain Array (Panomics) was performed according to the manufacturers’ instructions. For hybridization of whole cell lysates, cells were harvested as described above. Lysates were then incubated with 5 µg anti–Apo1 antibody and subsequently hybridized to the SH2-array membrane. The membranes were subsequently incubated with anti-CD95 antibody (C-20, Santa Cruz). After washing the array, it was incubated with streptavidin-HRP and developed.

**Bioluminescence imaging**

On day 21, the mice were injected intraperitoneally (i.p.) with luciferin (150 μg/g bodyweight) and placed on the in vivo imaging system (IVIS100; Xenogen). To prove whether the mice developed also metastasis in the liver and the lung, they were sacrificed 5 min after the administration of luciferin and the organs were measured ex vivo. Tumours, lungs and livers were measured ex vivo to increase measurement precision.

The bioluminescence signals were monitored at 10-s time intervals 5 min after the luciferin administration. The signal intensity was quantified as the sum of all detected photon counts within the region of interest after subtraction of the measured background luminescence.

**Supplementary Figure Legends**

**Supplementary Figure 1: Characterization of PDAC TICs**

(a) PanD3 and (b) PanD24 PDAC-TIC cells were incubated with the indicated concentrations of CD95L-T4, staurosporin (St., 1 µM) and gemcitabine (Gem, or left untreated (Co). After 24 h DNA fragmentation was analyzed by FACS. Results are expressed as means of biological duplicates ± SEM. (c) PET images of PanD24-derived tumours in three different mice 21 days after orthotopic injection of 10^4^ cells into the pancreatic tail of NOD-SCID mice. Tumour formation was confirmed by analysis using 18FDG. Coronal PET image shows hypermetabolic lesion (focus). (d) Freshly isolated PDAC cells from two independent surgical specimens (PanD80 and PanD82) were analyzed by flow cytometry for CD95 expression. CD45 staining was used to exclude immune cells. (e) Cell-surface CD95 expression was detected with FITC-labeled anti-human CD95 antibody and depicted as a histogram.

**Supplementary Figure 2: Human CD95L activates PI3K signaling cascade via the phosphorylation of Akt and ERK in established and primary PDAC cells**

(a) Phosphorylation of GSK upon treatment with the indicated doses of CD95L-T4 for the different time points is shown in PANC-1 cells. The lower panel shows the quantification of the P-GSK levels in PANC-1 cells as arbitrary units normalized to the untreated control (Co). (b) Phosphorylation of GSK upon treatment with the indicated doses of CD95L-T4 for the different time points is shown in PanD24 cells. The lower panel shows the quantification of the P-GSK levels in PanD24 cells as arbitrary units normalized to the untreated control (Co). (c) SH2 protein array performed with lysates from PanD3 cells.

(d) Knock-down efficiency on mRNA level for K-Ras by quantitative Real Time PCR. The values are normalized to control levels. Data correspond to Supplementary Figure 3d. Co: untreated cells; P: phosphorylated; T: total; KD: knock-down. (e) Knock-down efficiency on mRNA level for Sck by quantitative Real Time PCR. The values are normalized to control levels. Data correspond to Figure 5e and f. (f) Knock-down efficiency on mRNA level for CD95 and Sck by quantitative Real Time PCR. The values are normalized to control levels. Data correspond to Figure 6c. Co: untreated cells; P: phosphorylated; T: total; KD: knock-down.

**Supplementary Figure 3: Quantification of Western Blots from Figure 4**

(a and b) Quantification of the Western Blots in Figure 5a and b. Shown for all panels are relative values normalized to the untreated control (Co) in arbitrary units. (c) Phosphorylation of AKT and ERK upon treatment with CD95L-T4 is shown in PANC-1 cells at the indicated time points in cells transfected with non-targeting siRNA (Scrm) or siRNA targeting FADD transcripts (FADD KD). (d) Phosphorylation of AKT but not ERK upon transient transfection with non-targeting siRNA (Scrm) or siRNA targeting K-Ras transcripts (K-Ras KD) in PANC-1 cells. (e and f) Quantification of the Western Blots in Figure 5d and e.

**Supplementary Figure 4: Western blot analysis of EMT related factors after CD95 stimulation**.

(a and b) Western blot analysis of E-cadherin and Vimentin levels in PANC-1 and PanD24 cells after activation of the CD95 pathway. (c) Validation of anti-N-cadherin and anti-Fibronectin antibodies. * denotes respective proteins. (d) Comparison of E-cadherin and Vimentin levels between PANC-1 and PanD24 cells. (e) Western blot analysis of E-cadherin and Vimentin levels in PANC-1 cells after SCK knock-down. (f) SCK knock-down validation on mRNA level quantitative Real Time PCR.

**Supplementary Figure 5: Validation of CD95/CD95L antibodies for immunohistochemistry**.

(a) Paraffin sections of human colon epithelium (i), colon crypts (ii) and tonsil tissue (iii and iv) stained for CD95 (b) CD95L protein detection in epithelial tissue of paraffin embedded tonsil tissue using G247-4 (i and ii) and Abcam ab15285 (iii and iv) antibodies (Magnification: 400x).
