# Supplementary figures and images for: CD95 promotes metastatic spread via Sck in pancreatic ductal adenocarcinoma
Source: Cell Death Differ. 2015 Jan 23;22(7):1192–202. doi: 10.1038/cdd.2014.217 (PMC4572867; doi:10.1038/cdd.2014.217)

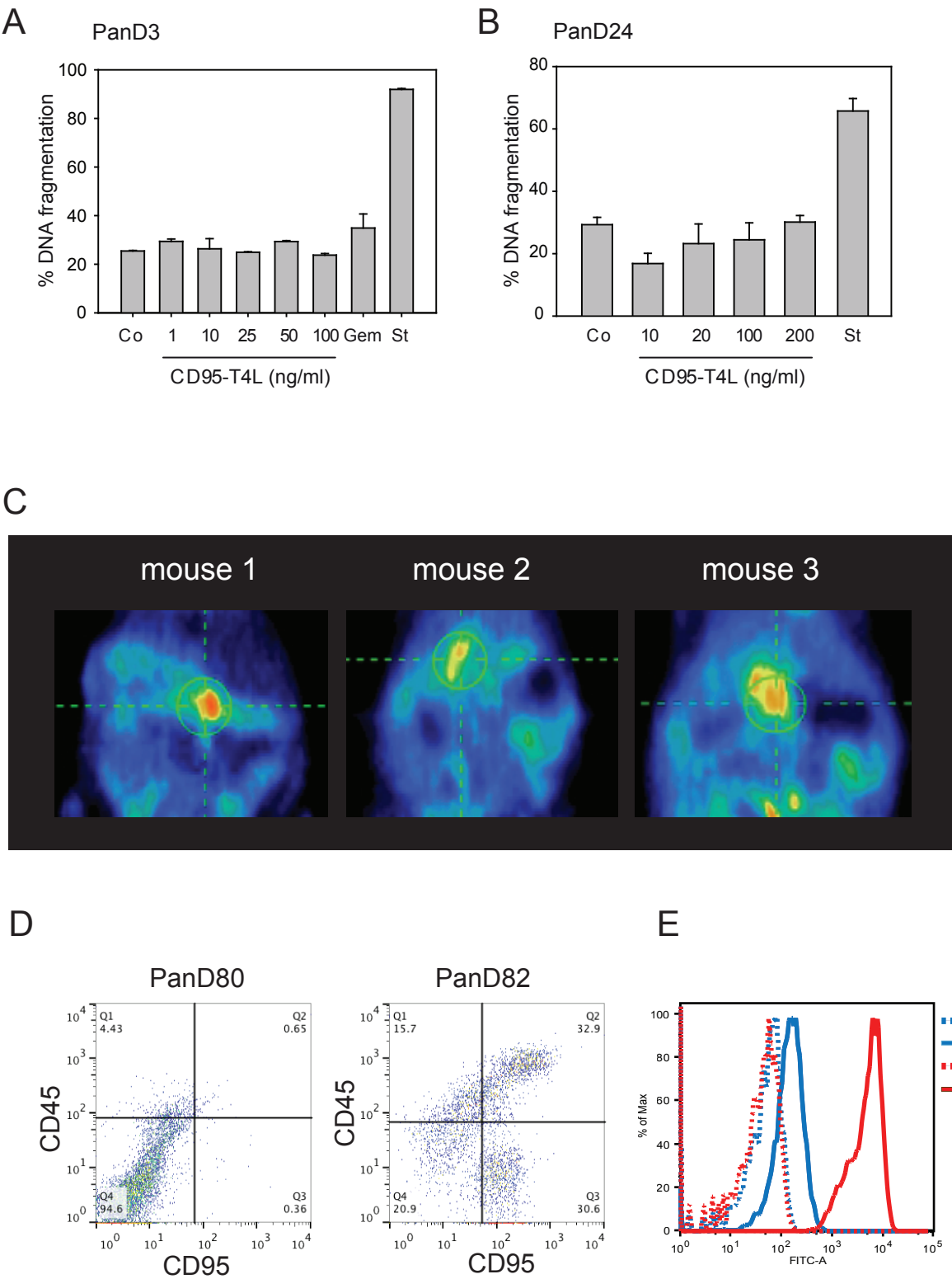

Supplement: Supplementary Figure S1 [file cdd2014217x2.pdf]

# Supplementary Figure 2

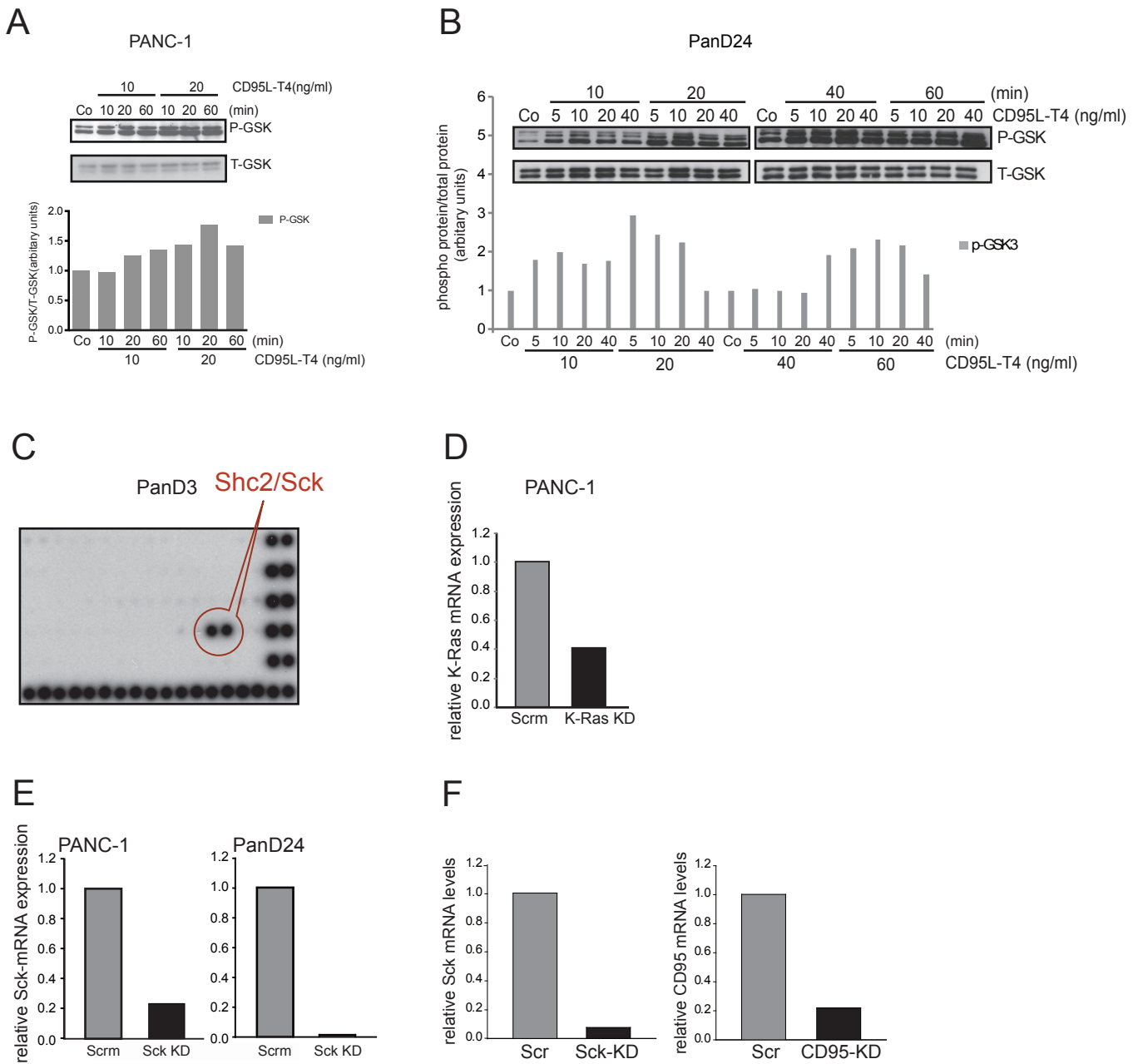

Supplement: Supplementary Figure S2 [file cdd2014217x3.pdf]

# Supplementary Figure 3

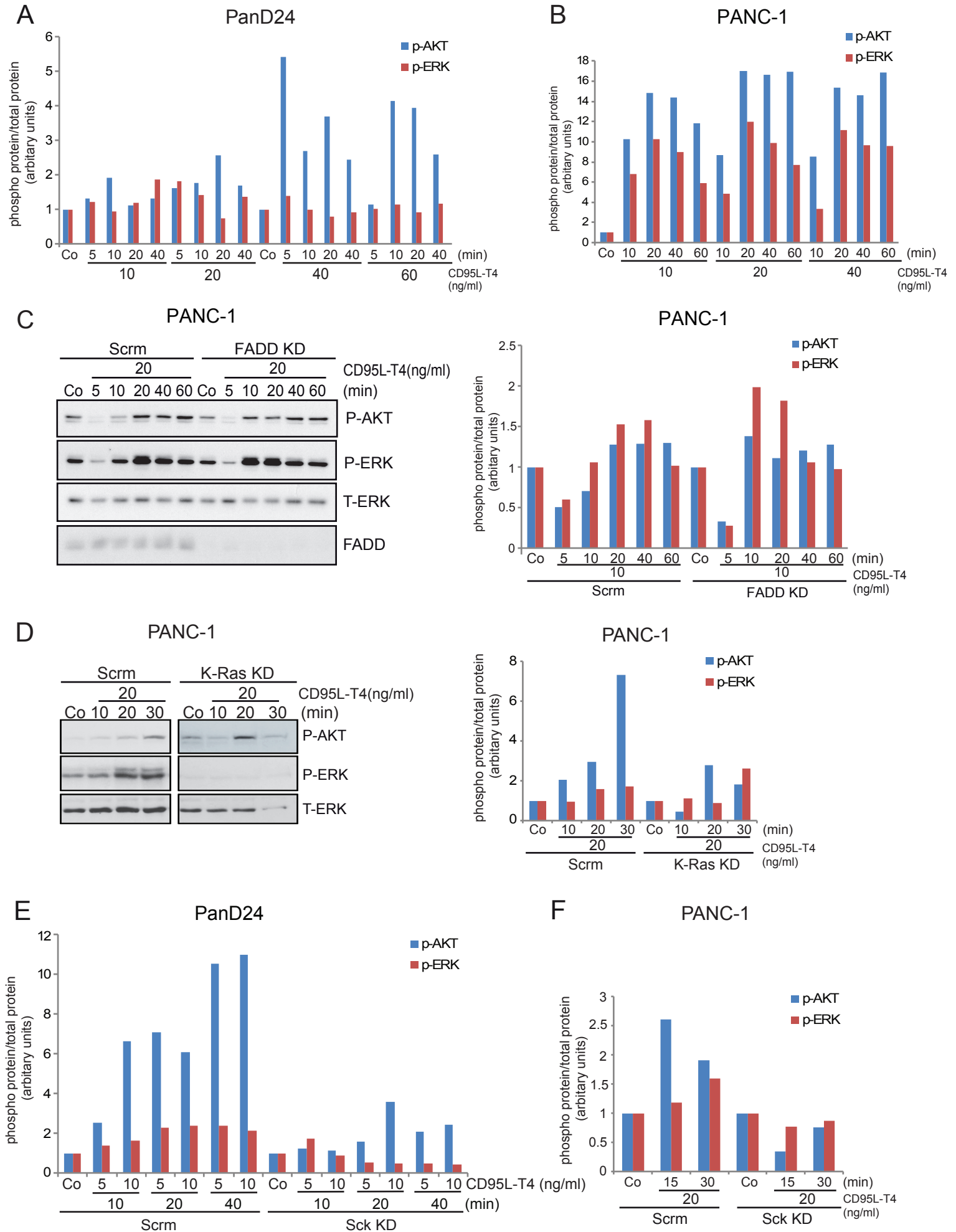

Supplement: Supplementary Figure S3 [file cdd2014217x4.pdf]

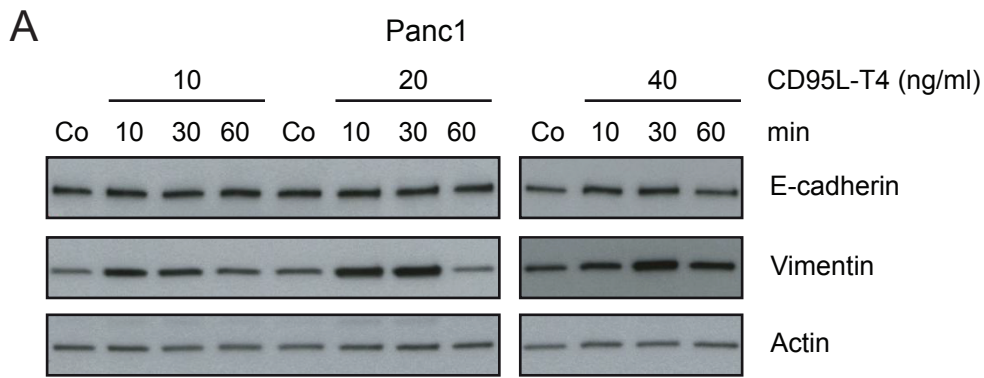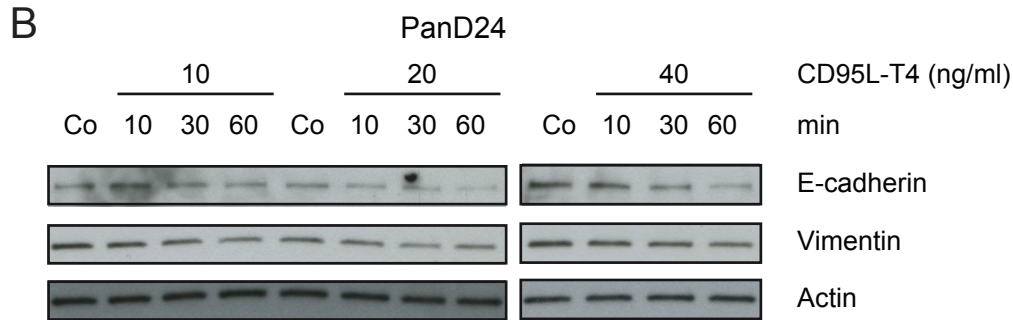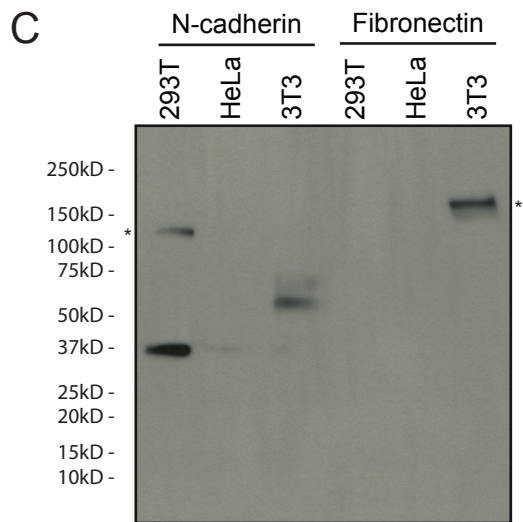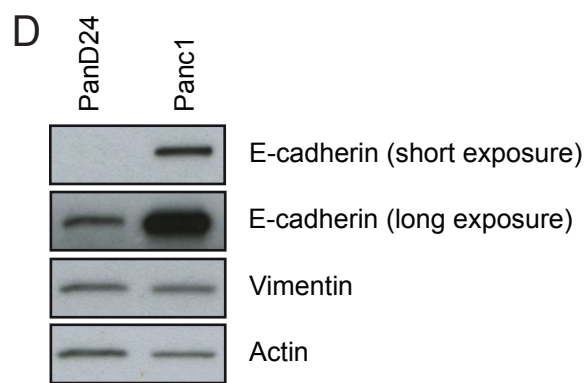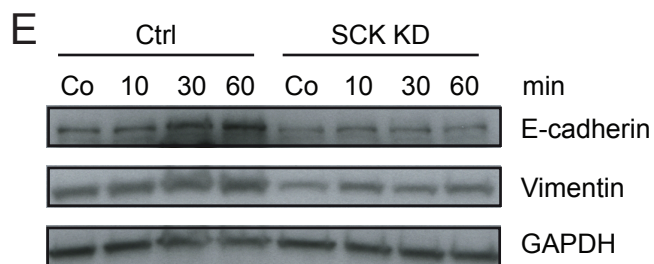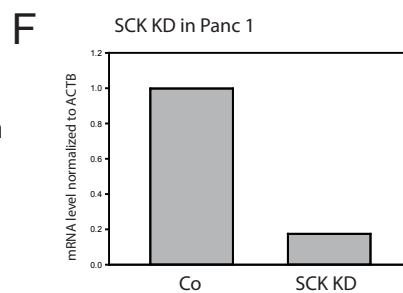

Supplement: Supplementary Figure S4 [file cdd2014217x5.pdf]

A

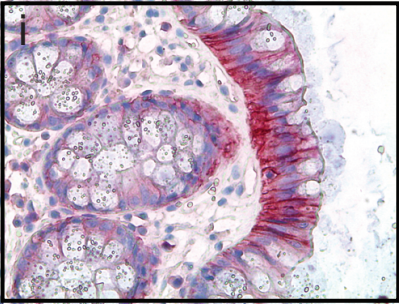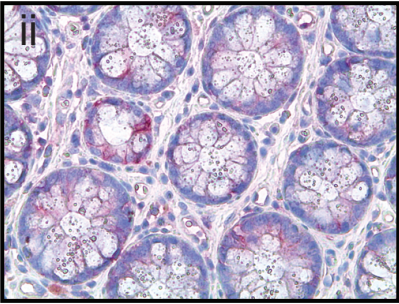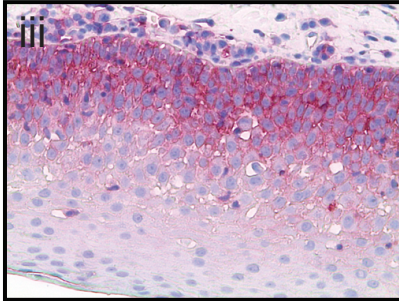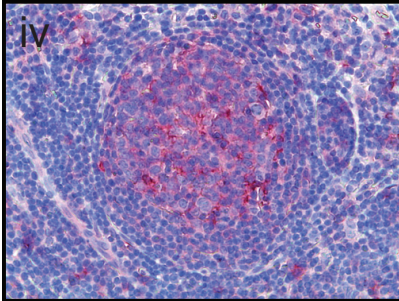

B

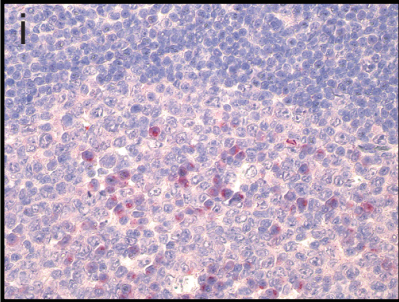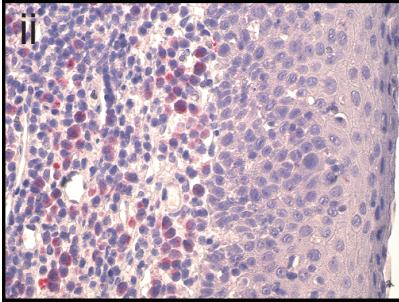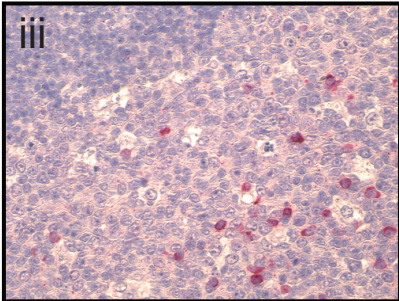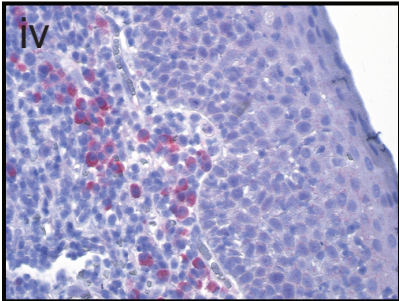

Supplement: Supplementary Figure S5 [file cdd2014217x6.pdf]
