# Supplementary material for: CD95 promotes metastatic spread via Sck in pancreatic ductal adenocarcinoma
Source: Cell Death Differ. 2015 Jan 23;22(7):1192–202. doi: 10.1038/cdd.2014.217 (PMC4572867; doi:10.1038/cdd.2014.217)
Supplement: Supplementary Table S1 [file cdd2014217x7.doc]

Table S1 Patient Diagnosis

| **Patient ID** | **Age** | **Sex** | **Stage (TMN)** | **Grade** | **Pathological Diagnosis** |
| --- | --- | --- | --- | --- | --- |
| A | 45 | Male | T3 N1 Mx | G3 | Pancreatic ductal adenocarcinoma |
| B | 62 | Male | T3 N1 M0 | G3 | Pancreatic ductal adenocarcinoma |
| C | 48 | Male | T3 N1 Mx | G3 | Pancreatic ductal adenocarcinoma |
| D | 37 | Female | T2 N1 M0 | G2 | Pancreatic ductal adenocarcinoma |
